# The influence of healthcare financing on cardiovascular disease prevention in people living with HIV

**DOI:** 10.1186/s12889-020-09896-8

**Published:** 2020-11-23

**Authors:** Allison R. Webel, Julie Schexnayder, C. Robin Rentrope, Hayden B. Bosworth, Corrilynn O. Hileman, Nwora Lance Okeke, Rajesh Vedanthan, Chris T. Longenecker

**Affiliations:** 1 Frances Payne Bolton School of Nursing, Case Western Reserve University, Cleveland, OH USA; 2 Duke University School of Medicine, Durham, NC USA; 3 Case Western Reserve University School of Medicine, Cleveland, OH USA; 4 The MetroHealth System, Cleveland, OH USA; 5 New York University Grossman School of Medicine, New York, NY USA

**Keywords:** HIV, Cardiovascular disease, Healthcare financing, Prevention, Relative value scales, Health planning support

## Abstract

**Background:**

People living with HIV are diagnosed with age-related chronic health conditions, including cardiovascular disease, at higher than expected rates. Medical management of these chronic health conditions frequently occur in HIV specialty clinics by providers trained in general internal medicine, family medicine, or infectious disease. In recent years, changes in the healthcare financing for people living with HIV in the U.S. has been dynamic due to changes in the Affordable Care Act. There is little evidence examining how healthcare financing characteristics shape primary and secondary cardiovascular disease prevention among people living with HIV. Our objective was to examine the perspectives of people living with HIV and their healthcare providers on how healthcare financing influences cardiovascular disease prevention.

**Methods:**

As part of the EXTRA-CVD study, we conducted in-depth, semi-structured interviews with 51 people living with HIV and 34 multidisciplinary healthcare providers and at three U.S. HIV clinics in Ohio and North Carolina from October 2018 to March 2019. Thematic analysis using Template Analysis techniques was used to examine healthcare financing barriers and enablers of cardiovascular disease prevention in people living with HIV.

**Results:**

Three themes emerged across sites and disciplines (1): healthcare payers substantially shape preventative cardiovascular care in HIV clinics (2); physician compensation tied to relative value units disincentivizes cardiovascular disease prevention efforts by HIV providers; and (3) grant-based services enable tailored cardiovascular disease prevention, but sustainability is limited by sponsor priorities.

**Conclusions:**

With HIV now a chronic disease, there is a growing need for HIV-specific cardiovascular disease prevention; however, healthcare financing complicates effective delivery of this preventative care. It is important to understand the effects of evolving payer models on patient and healthcare provider behavior. Additional systematic investigation of these models will help HIV specialty clinics implement cardiovascular disease prevention within a dynamic reimbursement landscape.

**Trial registration:**

Clinical Trial Registration Number: NCT03643705.

## Background

For many people living with HIV (PLWH), the scale-up of effective antiretroviral therapy has transformed a once fatal disease into a chronic condition. As PLWH are now living into their 8th and 9th decades, these individuals face increasing rates of other prevalent age-related chronic health conditions including cardiovascular disease (CVD). PLWH have a two-fold risk of developing CVD and experiencing an acute cardiac event compared to those without HIV [1]. While some of this excess cardiovascular risk is due to HIV-related factors (e.g., inflammation, HIV medication), traditional risk factors including hypertension, hyperlipidemia, obesity, and smoking also confer considerable risk [2]. Evidence-based strategies to reduce CVD risk are well-known, but are implemented at lower levels in HIV clinics [3].

Due in part to the stigma and fear associated with HIV infection early in the epidemic, HIV specialty clinics were created to provide compassionate, discrete care for PLWH. HIV specialists, who, over the course of this epidemic, have developed strong, trusting relationships with their patients, provide much of the HIV care in high-resource settings [4, 5]. As HIV care has evolved into chronic disease management, HIV providers have increasingly taken on the role of primary care physicians and are now managing the growing number of comorbidities. A majority of PLWH prefer to receive all health care in their HIV specialty clinic and visit their provider 3–4 times annually [6]. In the United States, healthcare is financed by a fragmented patchwork of payers including private employer-sponsored insurers, publically-funded Medicare and Medicaid for retirees and those with low-income, charity care for those who lack sufficient insurance, and the federally-funded Ryan White HIV/AIDS Program.

**Fig. 1:**
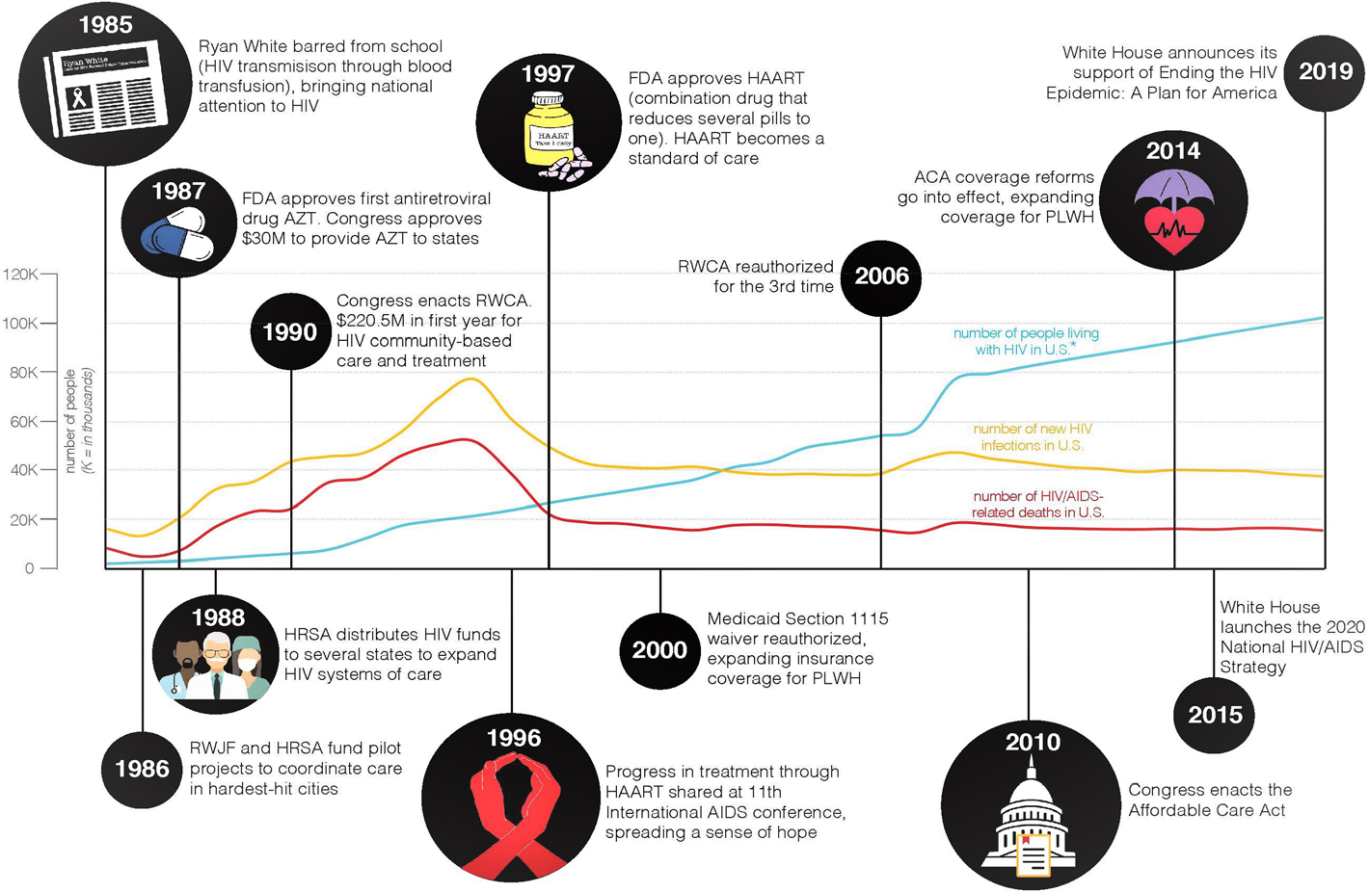
Timeline of HIV Healthcare Financing in the United States

Designed as a safety net program, the Ryan White HIV/AIDS Program (including the AIDS Drug Assistance Program (ADAP)) funds HIV clinics for medication, healthcare delivery, and wraparound services for uninsured and underinsured PLWH [7]. Originally authorized in 1990 (Fig. 1), today more than half of all PLWH in the United States receive services from this program each year [8]. The scope of services covered has also transitioned from HIV/AIDS care to encompass a larger array of primary health care services (e.g., screenings and immunizations) [9], yet for many HIV clinics CVD prevention remains limited. Therefore, there exists a unique, yet underutilized, opportunity to provide high-quality, consistent CVD prevention for PLWH.

In the general population, healthcare payers can influence the provision of preventative services by clinical providers and the adoption of preventive behaviors by patients. Initiatives to increase payments for cardiovascular quality measures and performance in the primary care setting, rather than solely for services rendered, have demonstrated reductions in hypertension, obesity, and blood glucose [10, 11]. This work indicates that no matter how it is paid for, CVD prevention requires tailoring at clinic and patient levels in order to be effective [10]. As the largest provider of HIV/AIDS services in the U.S., the Ryan White HIV/AIDS program has significant influence on HIV care, yet for CVD prevention, there are only two performance indicators that they measure- annual lipid screening and tobacco use screening and cessation [9]. Many of its performance indicators are understandably focused on HIV prevention and treatment [9]. However, such a singular focus may inadvertently minimize CVD prevention in this high-risk population. Furthermore, there is a significant gap in our understanding of how various healthcare payers influence CVD prevention in PLWH today. Our objective was to examine the perspectives of PLWH and their healthcare providers on how healthcare financing influences CVD prevention provided in HIV and primary care clinics.

## Methods

### Parent study

This qualitative analysis was drawn from the formative evaluation of CVD prevention in the Nurse-led Intervention to Extend the HIV Treatment Cascade for CVD Prevention (EXTRA-CVD) study. The EXTRA-CVD study is a randomized clinical effectiveness trial testing the efficacy of a multi-component, nurse-led intervention to reduce hypertension and high cholesterol in adults living with HIV [12]. It is part of the NHLBI-funded PRECluDE initiative dedicated to catalyzing the implementation of effective interventions for co-occurring CVD and lung diseases among PLWH [13]. The findings are reported according to the framework described in the Consolidated criteria for reporting qualitative research (COREQ) [14].

### Setting and sample

Fifty-one adults living with HIV and 34 multidisciplinary healthcare providers at three academic medical centers providing HIV specialty care in Durham, North Carolina (Duke Health) and Cleveland, Ohio (University Hospitals and MetroHealth) participated in this study. Participants were purposively recruited by telephone or mail from lists provided by the clinic liaisons, from the clinics in which they received healthcare or worked. PLWH were eligible if they were 18 years of age or older; received care at a participating HIV clinic; had a recent HIV viral load that was < 200 copies/ml; had hypertension (systolic blood pressure > 130 mmHg twice in the past 12 months and/or were taking anti-hypertensive medication); and had hypercholesterolemia (defined as a non-HDL cholesterol > 130 mg/dL or on cholesterol-lowering medication). Healthcare providers were eligible if they provided HIV care as a physician, nurse, social worker or medical assistant in the participating HIV clinic. Data were collected either in the clinic or by telephone.

### Data collection

After completing written informed consent, participants filled out a brief demographic survey. All participants completed either an interview (*N* = 48; 34 healthcare providers (HCPs) and 14 PLWH) or focus group (*N* = 6 focus groups; 37 PLWH) to elicit their perspectives on the facilitators and barriers of CVD prevention in HIV specialty clinics. Focus groups were preferred for PLWH because interaction among participants around the topics was thought to allow for more rich data to emerge. Additional interviews were added for PLWH out of a desire not to exclude individual PLWH who may represent an important population (e.g., those in rural settings or those without access to stable transportation). All interviewers (AW, JS or IS) were female, HIV-uninfected, had graduate-level training in qualitative interviewing, and were employed by academic centers in research roles. Two researchers had no prior relationships with participants (JS and IS) but one (AW) had worked with some of the HCPs on other research studies.

After an introduction in which the interviewer explained the study and her experience and interest in the topic, participants were encouraged to introduce themselves in a non-identifiable way (e.g., “Hi, I’m Mickey Mouse”). Focus groups and interviews were directed by one of three interview guides (Supplemental Material), to allow for tailoring of questions to PLWH, prescribing healthcare providers, and other members of the HIV care team (e.g., registered nurses, pharmacists). Interview questions were based on a literature search and focused on understanding the facilitators and barriers to high-quality CVD prevention care [6]. Study team members conducted interviews and focus groups in a private setting in the clinic between October 2018 and January 2019. Focus groups lasted approximately 60 min with between four and nine PLWH. All healthcare providers and those declining to participate in focus groups completed a face-to-face or telephone interview, which lasted approximately 30 min. Focus groups and interviews were digitally recorded and professionally transcribed verbatim; however, for the purpose of presentation, all quotes in this manuscript are reported in a naturalized transcription [15]. All procedures were approved by the University Hospitals, Cleveland Medical Center IRB and by reliant review at Metrohealth System and Duke Health.

### Data analysis

Descriptive quantitative data were managed using the Research Electronic Data Capture (i.e., REDCap) data management system [16]. All data were dual-entered and analyzed using appropriate measures of central tendency and dispersion.

Transcripts were managed using Dedoose Version 8.0.35 [17]. All transcripts were de-identified prior to analysis. To verify quality of transcription, 25% of all transcripts were reviewed for accuracy. Qualitative data were analyzed using template analysis - a structured form of thematic analysis with seven stages: data familiarization, preliminary coding, organizing preliminary themes, defining the coding template, applying and modifying the coding template, finalizing the coding template, and data interpretation [18]. Consistent with the purpose of the EXTRA-CVD study to understand the local clinical context in which CVD preventative care occurs in HIV settings [19], the code tree consisted of 14 domains of the theoretical domains framework. All transcripts were then independently coded by two members of the study team (J.S. and A.W.) who met regularly to review and assign final codes. During these meetings study team members also examined how their prior work in the field, or their clinical experience, may influence their interpretation of the data. While none of the interviewers worked clinically alongside the HCPs or delivered care to the PLWH enrolled in this study, AW and JS had worked extensively in HIV research and clinical care and during these discussions considered how this prior work may have influenced data analysis and interpretation. Additionally, during these meetings coding disagreements were resolved through consensus.

To answer the question “How does healthcare financing influence CVD prevention in PLWH?” we conducted a thematic analysis of the subset of the interview and focus group content associated with the Environmental Context and Resources Domain (838 excerpts) [20]. This domain encompasses the resources and material resources, environmental stressors, person and environment interactions, and knowledge of the task environment as they pertain to the context in which CVD preventative care is delivered to PLWH. The team reviewed themes within this code for relevance and determined the best fitting themes associated with two or more sites. All transcripts were re-reviewed to verify the presence, fit, and depth of content associated with each retained theme. Final themes were present if appearing in transcripts from two or more study sites and further analysis yielded no new information or changes to the codebook [21]. The meaning of these themes in relation to the research question was similarly discussed among all team members and representatives (e.g., HCPs and PLWH) from each of the clinical sites during meetings to adapt the EXTRA-CVD intervention to each clinic setting [22]. Data that best exemplified these themes are included in this manuscript.

## Results

Across all sites, a total of 51 PLWH and 34 healthcare providers participated in the study (Table 1). Thirty-four (67%) of the PLWH and 11 (32%) of the healthcare providers were male. The majority 36 (71%) of PLWH identified as African American and 24 (74%) of HCPs identified as white. Almost half (44%) of the healthcare providers were physicians and 24% were registered nurses. On average, PLWH had been living with HIV for 19.6 years and 94% reported receiving primarily public insurance (e.g., Medicare, Medicaid, or Ryan White/ADAP). Several PLWH declined to participate in focus groups due to privacy and time concerns but agreed to be interviewed by phone.

**Table 1:** Demographic Characteristics of All Participants, by Site

	Site 1		Site 2		Site 3	
	PLWH^a^ *n* = 16	HCPs^b^ *n* = 10	PLWH *n* = 17	HCPs *n* = 12	PLWH *n* = 18	HCPs *n* = 12
Sex						
Male (%)	11 (68)	2 (20)	9 (53)	4 (33)	14 (78)	5 (42)
Female (%)	5 (31)	8 (80)	8 (47)	8 (67)	4 (22)	7 (58)
Race						
Black/African American	12 (75)	2 (20)	11 (65)	0	13 (72)	2 (17)
White/Caucasian	4 (25)	7 (70)	3 (18)	11 (92)	5 (28)	7 (58)
Multiple/Other	0	1 (10)	3 (18)	1 (8)	0	3 (25)
Education						
11th Grade or less	1 (6)	0	5 (29)	0	0	0
High School or GED^c^	5 (31)	0	3 (18)	0	4 (22)	0
Some College	7 (44)	3 (30)	7 (41)	3 (25)	9 (50)	1 (8)
Bachelor’s or Masters Degree	3 (19)	3 (30)	2 (12)	3 (25)	5 (28)	5 (42)
Doctorate	0	4 (40)	0	6 (50)	0	6 (50)
Health Care Provider Specialty						
Licensed Social Worker		1 (10)		3 (25)		2 (17)
Registered Nurse		3 (30)		3 (25)		2 (17)
Physician		3 (30)		6 (50)		6 (50)
Other		3 (30)		0		2 (17)
Insurance Type of PLWH						
Public	13 (81)		17 (100)		18 (100)	
Private	3 (19)		0		0	
Mean Years since HIV Diagnosis (±SD)^d^	20.8		14.7		19.6	

*Abbreviations*: ^*a*^*PLWH* People Living with HIV, ^*b*^*HCP* Healthcare Providers, ^*c*^*GED* General Education Development Test, ^*d*^*SD* Standard Deviation

Analysis of the qualitative data addressing the question, “How does healthcare financing influence CVD prevention in PLWH?” revealed several themes. First, the data suggest that health insurance payers have substantial control over decisions affecting the cardiovascular care and treatment of PLWH. Second, health systems—including physician compensation plans—organized around relative value units (RVUs) disincentivize CVD prevention efforts by HIV specialty care providers. Finally, there was evidence that grant-based services enable locally-tailored CVD prevention strategies but the scope and reach of these services are limited by the sponsor’s priorities (Table 2).

**Table 2:** Themes Describing how Payers Influence Cardiovascular Preventative Care in HIV

Theme	Description	Patient Quote	Provider Quote	Implications for HIV Primary Care
Power of the Purse	Payers exert significant control over the in clinic delivery of cardiovascular preventative care and the at-home self management of preventative behaviors	“I moved here three and a half years ago. Previously, my HIV doctor was also my primary doctor. The insurance systems out there allowed that. When I moved here, the insurance systems don’t allow that. They force you – my HIV doctor was going to be my primary, but the insurance coverage forced it that I had to have a primary.” – Male PLWH, 24 years living with HIV “Cost can be a factor [in taking cardiovascular medications] and fighting the insurance company can be a factor.” – Female PLWH, 17 years living with HIV	“Oh, insurance. No. 1. Insurance. Insurance companies make it very difficult. I think they try, when they’re trying to tell you what’s better for the patient rather than the doctor dictating it. That’s frustrating” –Female Social Worker, 6 years HIV experience	• Clinics receiving Ryan White HIV/AIDS Program funds may need additional interventions to increase awareness about the importance of cardiovascular disease prevention in HIV • Enhanced communication between commercial insurers, patients, and clinical staff may result in more effective implementation prevention strategies • Expanded access to cardiovascular disease prevention tools for PLWH can help mitigate their increased risk • HIV and primary care providers should consider working together to develop new models of value-added care tailored to the needs of PLWH
Relative Value Units pressures may disincentive CVD Prevention	Health systems organized around relative value units (RVUs) experience pressures that may disincentivize CVD prevention efforts by HIV specialty care provider		“And so, things that make our job more difficult, I think are the sort of business side of medicine that most people recognize throughout the world, or at least the United States, that is the bureaucracy” –Male Physician, 20 years HIV experience	• Deem a higher value for comprehensive prevention services provided by the HIV team • Stronger consideration of bundled preventative service, perhaps through the annual wellness visits, in HIV clinics • Consider de-emphasizing and de-coupling RVU generation from providers’ annual performance
Grant funding can be a double-edge sword in CVD prevention	Grant-based services enable locally-tailored CVD prevention strategies but are limited by the funder’s timeline, priorities and requirements	“We do have other groups here. You ask your doctor and they can give you instructions. We used to have X. It used to be on Main Street. But the one here in closed.” - Male PLWH, 6 years living with HIV	“Because one of the priorities in quality improvement that our funders have seen is that youth aren’t getting in to – they’re not getting virally suppressed, so they’re not getting into care, taking their meds, they’re the vulnerable population right now.” Female Social Worker, 4 years HIV experience	• Holistic evidence-based cardiovascular health initiatives can be better integrated, and consistently funded, into HIV clinics • Proposed sustainability plans should include how the team will engage with payers to facilitate the long-term integration of prevention initiatives

### Power of the purse: Insurance controls CVD decision making

Health insurance provides payment for services by spreading costs throughout a risk pool and the power insurers have to control access to CVD prevention services and resources was evident to both healthcare providers and patients. This was a dominant theme. Many healthcare providers offered experiences demonstrating how insurance influences, and sometimes hinders, the provision of necessary resources for their patients. Beyond just paying for services, through their annual performance measures the Ryan White HIV/AIDS Program also signifies to healthcare providers which healthcare services are important for PLWH.

“I wouldn’t even know where to get a blood pressure (cuff) – I mean, would they have to pay for a monitor and how would we get it to them? Would it be covered? A lot of my patients get their medications through ADAP (AIDS Drug Assistance Program), and not a lot of non-HIV medication, so this not being an HIV-related thing, it probably wouldn’t be covered.” - Female Physician, 12 years HIV experience

“And then, insurance wouldn’t cover the smoking cessation thing because some patients would come in, ‘Well, my insurance said they won’t pay for it, and I can’t afford it on my own,’ and that’s when they started smoking again.” - Female Medical Assistant, 3 years HIV experience

Patients described how insurance regulations are not tailored for PLWH who are at increased risk for CVD. Many PLWH trust their HIV provider to manage all aspects of their health care, including their cardiovascular care. Yet, insurance companies had the authority to dictate and disrupt that relationship.

“I think the problem is, when you get these HMOs, like X, they will tell you that you need a family medicine doctor as your primary care doctor. These insurance companies won’t tell you that you can petition them to have your ID [infectious disease] doctor as your primary care physician. So, some people get caught in having that other primary care doctor that really is doing them no good, so. So, I think it impacts overall health.”

- Male PLWH, 19 years living with HIV

“You know what my shrink told me? She was going over my med list she said, ‘You know, you can’t take these two together.’ I’m like, ‘No,’ she said, ‘Do you have muscle cramps?’ ‘All the time!’ she said, ‘They’re interacting,’ she said, but I can’t get rid of the blood thinner because the insurance company doesn’t want to pay for nothing” - Male PLWH, 29 years living with HIV

### Relative value units (RVUs) pressures may Disincentivize cardiovascular disease prevention

Medicare reimburses healthcare providers for their services based on relative value units (RVUs), which is a rank of the resources used to provide the service on a common scale [23]. Healthcare systems rely on these payments to support not only providers’ salaries but also the support staff and additional resources necessary for the provision of healthcare. Some healthcare systems tie a provider’s annual performance evaluation to the RVUs they generate, increasing patient volume pressures, which may result in less time spent with each patient. The primacy of RVUs in the larger business of healthcare was evident in the healthcare provider interviews.

“I’m sure, in every healthcare institution, the resources are limited. There are so many patients, and there’s so much emphasis on RVUs, and there’s not much staffing. So, there’s resistance to change, which is sad. But something like integrated HIV and CVD care would be so helpful for patients.” Male Physician, 20 years HIV experience

While they may not have heard of RVUs, patients felt the same time pressures. Their healthcare team was often too busy to provide additional counseling around CVD prevention and treatment. This limited time with their provider affected their knowledge and engagement in CVD self-management.

“I feel – I know the doctors are always busy. But it seems like there should be some time that we could just have a room, even if each one of us could have our own doctors be there just for a little while. At least about an hour, we would really interact and ask questions, because we can’t. We don’t get nothing but 15 minutes.” – Female PLWH, 20 years living with HIV

In general, both the healthcare providers and the PLWH felt like these time and resource pressures limited the cardiovascular health promotion among PLWH and were frustrated with the current system.

### The double-edged sword of Grant funding

The final theme illustrating the influence that healthcare financing exerts on CVD prevention was the double-edged sword of grant funding. Grants offer less restrictive funding to fill service gaps for HIV prevention and community-based care. At the same time, grant funders often require full distribution of awards within specific, time-bound reporting and also require performance metrics that can burden clinic capacity. Healthcare providers described how various types of grant funding augmented the cardiovascular services they could typically provide.

“We try very hard to plan our soft-money projects based on larger clinic needs and to take that into account together in terms of how we can fund staff and get what we need for our patients.” - Female Social Worker, 29 years HIV experience

Successful implementation of programmatic grant funding requires champions. Within the HIV clinics, cardiovascular initiatives were championed by nurses, social workers, physicians, and nutritionists depending on the staff’s interests. Champions developed the concepts, fostered staff motivation, obtained funding, and helped with the reporting and regulatory work.

“Healthy Harvest was expanding. I knew about it and I was like, ‘Well, why can’t we bring that to our [HIV] clinic?’ Because we know food accessibility is an issue here, and they were providing bags of free produce, which our patients could benefit from. So, I reached out to the person who was in charge of that initiative. I’ve had the opportunity to make a garden for the patients in which we’re providing produce every week during the summer months. Twenty-pound bags of produce that our patients can take at no cost to them to help them improve their diet.” - Male Dietician, 3 years HIV experience

These programs offered providers flexibility in delivering resources and services to PLWH. Healthcare providers expressed satisfaction by being able to deliver these programs. Further, while this theme was mostly described by the providers, PLWH voiced support for the importance of the grant-funded programs mentioned (e.g. clinic-based food banks) to their cardiovascular health. Yet, relying on such programs is bounded by funder priorities and creates pressure, given that clinics have little control in aligning and adapting their missions and services to varying funder goals.

“Because one of the priorities that our funders have seen is that youth aren’t getting in to – they’re not getting virally suppressed, so they’re not getting into care, taking their meds, they’re the vulnerable population right now.” - Female Social Worker, 4 years HIV experience

The grant-funded programs were seen as mostly beneficial to participant’s cardiovascular health because they opened up new opportunities for patients but had several negative consequences (e.g., shaping the program to the funder’s priorities, increased workload) that may limit their impact.

## Discussion

Preventing CVD in PLWH is an emerging challenge with little data to guide policy development and implementation. The transition of HIV from a fatal to a chronic disease has occurred quickly and only recently have patients, healthcare providers, and healthcare organizations started to recognize this growing CVD burden. In the general population, payers harbor great influence on the delivery of CVD prevention [24], and our novel data indicate this is also true for PLWH who receive much of their care in specialty clinics. Specifically, our primary theme was that beyond the power payers have on dictating the scale at which services are funded, they also set the agenda for what is perceived to be important by PLWH and healthcare providers.

HIV specialty clinics have adeptly evolved in order to provide primary care and CVD prevention, which is quite different than their original focus on opportunistic infections and AIDS. The primary funding source of these clinics – the Ryan White HIV/AIDS Program – has been slower to evolve proactively to the growing CVD burden. While this program provides critical wraparound and medical services for PLWH, our data make it clear that this program may need to expand its services and evaluation metrics to encompass cardiovascular prevention. Doing this and clearly and consistently communicating these expanded services to HIV providers, will provide PLWH the resources to obtain evidence-based prevention services (e.g. home blood pressure monitoring, targeted cholesterol medications, coronary calcium scoring) [25]. It may also incentivize HIV clinicians who provide primary care to appropriately assess and manage cardiovascular risk. Our data also suggest that for those HIV providers who do not provide primary care, working closely with the patient’s primary care provider can strengthen their (primary care provider and PLWH) relationship, which may increase the patients’ prevention behaviors [26]. Several interventions designed to improve the relationship between primary and HIV care to reduce CVD are currently under investigation [13]. It is also possible that HIV and primary care teams can work together to develop a new model of value-added care for PLWH that synergistically improves both HIV and cardiovascular outcomes for this vulnerable population. Future work should build on these findings and examine how to best develop and test these new models of care.

The tension between encouraging timely, high-quality, innovative health care and containing costs is a longstanding challenge to American healthcare and these issues are not unique to PLWH. Understandably, healthcare administrators are continually trying to maximize fiscal performance and create operational efficiencies; however, the pressure to produce RVUs has been found to decrease healthcare provider satisfaction [27, 28] In response, some have advocated to update the RVU scale to thoughtfully incorporate more accurate measures of time and healthcare quality [29]. However, little published data could be found that integrated the economic consequences of RVU-based systems with the perspectives of healthcare providers and patients. Our findings that HIV providers think the emphasis on RVU generation limits the promotion of cardiovascular health in this high-risk population are novel and need further exploration.

To help balance the tension between costs and quality, there has been a growth in patient-centered medical homes which provide efficient and high-quality care [30]. While the HIV specialty clinics in our study share many similarities with patient-centered medical homes (e.g., patient-centered care, co-located services), they are not designated as such. Future research, conducted in real-world settings, should consider the established benefits of providing cardiovascular care in HIV specialty clinics (e.g., increased patient-provider trust, regular visits, perceptions of safe clinic space) [31] with the challenges of providing co-located care (e.g., medically complex patients, trained HIV specialists providing primary care, role of larger health care system policies). Minimally, these data suggest a new reimbursement and incentivizing plan may be needed in order to improve the cardiovascular health of PLWH as a population.

We also observed that grants may be a useful way to fund CVD prevention efforts for PLWH, often to specifically address the social determinants of health related to poverty and access to healthy lifestyles. Creating sustainable food banks, transportation, and social support networks are critical for the health of all. Yet most grant funding, whether from private foundations or federal grants, supports isolated projects that may or may not lead to sustainable improvements in the health of vulnerable populations [32]. These grants reflect the priorities of a single entity, and funds tend to be restricted and time-bound. Our findings, in the context of the larger literature, indicate that while grant-funded projects promote cardiovascular health, institutional support must be sustained beyond the length of a funded project in order to improve health outcomes [33]. This sustainability will require a clear integrated path for getting payers to finance these services, including those that mitigate social risk factors. One model may be the successful translation of the National Diabetes Prevention Program, a lifestyle intervention demonstrated to reduce disease and healthcare costs [11]. After decades of research and advocacy, this effective program is now covered by a wide-range of payers, including Medicare, helping to institutionalize its benefits.

In considering the different perspectives of HCPs and PLWH, it is clear that the healthcare financing barriers to CVD prevention care in PLWH is most strongly experienced by HIV care providers. Through PLWH offered data pertaining to how insurance companies impede CVD preventative measures, the data provided by HCPs had more emphasis and was more frequent. As one might expect, much of the data describing RVU pressures was described in detail by the HCPs but not by PLWH. In the U.S., patients are largely disconnected from the business of their healthcare financing and lack understanding of their provider’s incentives structure including RVUs [34]. Yet, as our data and that of others demonstrates, this incentive structure can influence the daily professional decisions and can have a dramatic impact on the health of individuals and populations. Finally, both HCPs and PLWH described a double-edge sword of grant funded programs. While less frequent than the other themes, it was much more consistent between both types of participants.

Finally, our findings are bolstered by a number of study strengths including: (1) a wide diversity of perspectives from different types of healthcare providers and PLWH from multiple sites; and (2) rigorous qualitative methods, including a large sample who provided sufficient data to reach saturation (i.e., when additional transcripts led to few changes in the codebook) [21] . The study may be limited by its purposive sampling methods, which may increase risk of bias but may also be helpful in obtaining a wide range of perspectives on the issues. Our interview guide was framed to help understand the facilitators and barriers to implementing high-quality CVD prevention care, and it did not contain a priori questions focused on health care financing systems. Rather, these data emerged inductively during conversations with the interviewer. This may have limited our ability to describe additional, relevant themes. Further, while we are able to make payer-related comparisons among PLWH given the variability in insurance benefit managers, many PLWH reported having public insurance, which may limit the transferability of these data.

## Conclusions

As healthcare financing for PLWH evolves, an understanding of the effects of various payers on patient and healthcare provider behavior as well as the responses of the healthcare systems in which this care is provided, is important. HIV specialty clinics are an ideal environment to integrate comprehensive CVD prevention strategies into everyday HIV care. To be sustainable though, these strategies must align with the dynamic reimbursement landscape. HIV clinics should also be at the forefront of advocating for healthcare delivery and reimbursement models responsive to the evolving medical needs of PLWH.

## Supplementary Information

**Additional file 1.** Consolidated criteria for reporting qualitative studies (COREQ): 32-item checklist.

## Data Availability

Qualitative datasets generated and analysed during the current study are not publicly available due to the potential for de-identification but are available from the corresponding author on reasonable request.

## Abbreviations

ADAP: AIDS Drug Assistance Program
CVD: Cardiovascular Disease
HDL: High Density Lipoprotein
HMO: Health Maintenance Organization
NHLBI: National Heart Lung and Blood Institute
PLWH: People living with HIV
RVU: Relative Value Units
